# Vasculogenic Mimicry in Breast Cancer: Clinical Relevance and Drivers

**DOI:** 10.3390/cells10071758

**Published:** 2021-07-12

**Authors:** Gabriela Morales-Guadarrama, Rocío García-Becerra, Edgar Armando Méndez-Pérez, Janice García-Quiroz, Euclides Avila, Lorenza Díaz

**Affiliations:** 1 Departamento de Biología de la Reproducción, Instituto Nacional de Ciencias Médicas y Nutrición Salvador Zubirán, Ciudad de México 14080, Mexico; gabriela.mguadarrama@gmail.com (G.M.-G.); edgar.mendez.p3@gmail.com (E.A.M.-P.); janice.garciaq@incmnsz.mx (J.G.-Q.); euclides.avilac@incmnsz.mx (E.A.); 2 Departamento de Biología Molecular y Biotecnología, Instituto de Investigaciones Biomédicas, Universidad Nacional Autónoma de México, Ciudad de México 04510, Mexico; rocio.garciab@iibiomedicas.unam.mx

**Keywords:** vasculogenic mimicry, breast cancer, tumor neovascularization, HER2, triple-negative

## Abstract

In solid tumors, vasculogenic mimicry (VM) is the formation of vascular structures by cancer cells, allowing to generate a channel-network able to transport blood and tumor cells. While angiogenesis is undertaken by endothelial cells, VM is assumed by cancer cells. Besides the participation of VM in tumor neovascularization, the clinical relevance of this process resides in its ability to favor metastasis and to drive resistance to antiangiogenic therapy. VM occurs in many tumor types, including breast cancer, where it has been associated with a more malignant phenotype, such as triple-negative and HER2-positive tumors. The latter may be explained by known drivers of VM, like hypoxia, TGFB, TWIST1, EPHA2, VEGF, matrix metalloproteinases, and other tumor microenvironment-derived factors, which altogether induce the transformation of tumor cells to a mesenchymal phenotype with a high expression rate of stemness markers. This review analyzes the current literature in the field, including the participation of some microRNAs and long noncoding RNAs in VM-regulation and tumorigenesis of breast cancer. Considering the clinical relevance of VM and its association with the tumor phenotype and clinicopathological parameters, further studies are granted to target VM in the clinic.

## 1. Introduction

### What Is Vascular Mimicry?

Tumor growth and dissemination depend on vascularization, a process that is achieved through vasculogenesis and/or angiogenesis. Vasculogenesis is de novo blood vessel formation by newly differentiated endothelial cells (ECs), while angiogenesis is the formation of blood vessels from pre-existing ones, either by sprouting or intussusception [1]. Intussusception refers to the formation of pillars inside the blood vessel, resulting in its division into segments [2]. In the context of cancer, both vasculogenesis and angiogenesis are regulated by microenvironment-derived factors, tumor heterogeneity, cell–cell interactions (including malignant and non-transformed cells), as well as modifications on extracellular matrix (ECM) components [3]. In addition, there are alternative non-angiogenic mechanisms used by tumor cells to obtain nutrients and oxygen and to disseminate to distant sites, for instance vessel co-option, which consists in the hijacking of pre-existing blood vessels from non-tumoral surrounding tissue [4]. While all these processes and their related pathways play an essential role in the growth, proliferation, migration, invasion, and metastasis of highly aggressive tumors, they are not the only mechanisms by which tumors generate vasculature and escape routes [5,6]. In 1999, Maniotis and collaborators described for the first time an endothelial-independent vascularization mechanism in highly aggressive and metastatic uveal and cutaneous melanoma tumors. They observed the presence of patterned networks of interconnected loops and cord formation, composed of cancer cells and ECM that stained with the periodic acid-Schiff (PAS) reagent, but that in relation to EC markers such as CD31 or factor VIII-related antigen, showed weak, focal, and discontinuous staining. Erythrocytes could be found inside these structures, suggesting that they actually conducted blood and represented an intratumor microcirculatory system. Moreover, it was shown that highly invasive M619 human melanoma cells were able to form three-dimensional channel-like structures resembling vascular networks. This process was termed “vasculogenic mimicry” (VM) [7,8,9,10].

Vascular channels in VM share several characteristics with endothelial-dependent vasculature; however, distinctive features differentiate them (Table 1). For instance, ECs express vascular endothelial (VE)-Cadherin, also known as CD144, the major molecule related to cell–cell adhesion in endothelial adherent junctions. However, in cancer cells capable of forming VM, VE-Cadherin is aberrantly expressed and seems to be involved in a different function, namely, the acquisition of tubule-like structures [11]. Even if at present time there is no infallible biomarker for VM channels identification, some specific characteristics and the expression of particular markers associated with these cellular arrangements have been described (Table 1).

**Figure 1:**
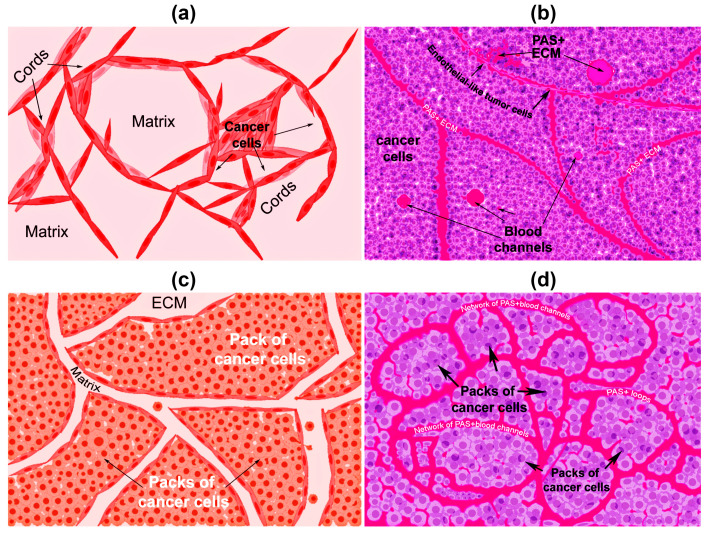
Graphical representation of the types of vasculogenic mimicry structures formed in vitro and in vivo by cancer cells. (**a**) In vitro tubular type. Is formed by networks of cellular cords encircling cell-free spaces above a matrix rich in collagen such as Matrigel. Tumor cells aligned in cords or tubular-like interconnected structures are depicted. (**b**) In vivo tubular type (parallel PAS+ patterned). Deposits of PAS+ proteoglycan/laminin-enriched matrix derived from cancer cells resembling “matrix rivers” may contain PAS+ tumor cells able to form channels and may be flanked by endothelial-like tumor cells. (**c**) In vitro patterned matrix type. Flattened tumor cells lodged into the matrix form packages of cells that deposit matrix enriched in collagen, laminin, and proteoglycans. Tumor endothelial-like cells may be found surrounding the packs. (**d**) In vivo patterned matrix type (network or back-to-back loops PAS+ patterned). Several layers of extracellular matrix rich in laminin, fibronectin, and collagens IV and VI form loops surrounding packs of tumor cells.

**Figure 2:**
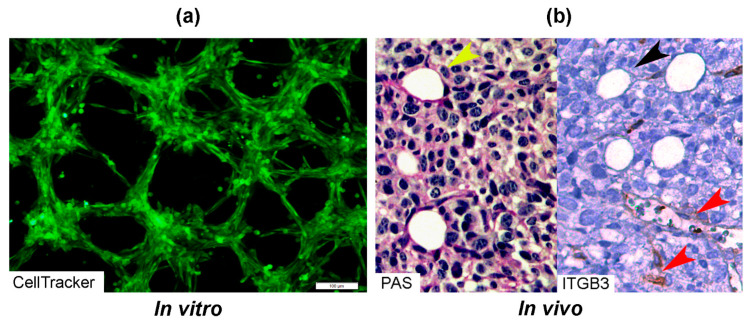
Photographs of vascular mimicry structures formed by the TNBC cell line MBCDF-Tum in vitro and in vivo. (**a**) MBCDF-Tum cells were labeled using Cell tracker-green (Abcam) and photographed by epifluorescence microscopy at 24 h of seeding (10 magnification, Olympus BX51). (**b**) MBCDF-Tum cells xenografted in nude mice generated VM-forming tumors. VM structures (yellow arrow heads) were identified by PAS-staining (magenta color, left side of the picture) in a tumor section. In the right part of the picture, a similar section of the same tumor was stained for Integrin-β3 (ITGB3) as an endothelial marker, identifying tumor endothelial-vasculature (brown staining, red arrow heads) in a hot spot of ITGB3-negative VM channels (black arrow heads) (40 magnification).

There are two types of VM described up to now. The tubular type and the patterned matrix type [22] (Figure 1). In vitro, the first type refers to networks of cellular cords above a thin matrix, encircling cell-free spaces (Figure 1a). In vivo, this type would appear as matrix “rivers” that may arrange as parallel PAS+ ECM deposits (Figure 1b). This matrix is produced by cancer cells. In some cases, PAS+ tumor endothelial-like cells can be found forming cords or lining blood channels. In the second type, PAS+ ECM patterns enclose packs of tumor cells wedged into the matrix arrays (Figure 1c,d). This last one is characteristic of highly invasive tumors [22]. It may be possible that the patterned type gives rise to the tubular type, after the enclosed cells die. In Figure 2, we provide photographs depicting VM-structures formed in vitro and in vivo by the triple-negative breast cancer (TNBC) cell line MBCDF-Tum, reported as highly tumorigenic [23] (Figure 2).

## 2. First Highlights of VM in Breast Cancer

VM has been identified in numerous types of highly aggressive tumors including breast cancer. Only two years later from the first report of VM in melanoma, a group in Japan identified the presence of blood pooling without a lining of ECs on hyper vascularized xenografts of inflammatory breast cancer. Remarkably, these cells were able to form tube-like structures and loops in vitro, and were associated with lung metastasis in vivo, representing the first evidence of VM in breast cancer [24]. These results helped to establish the relationship between angiogenesis and VM. Shirakawa et al. observed that the hyper vascularized zone in the tumor periphery contained vessels lined by ECs positive to murine CD31, consistent with angiogenesis, while the central highly hypoxic area of the tumor exhibited channels that were PAS positive, presented weak expression of human integrin α_v_β_3_ and lacked ECs, consistent with VM. Altogether, this suggested that in some instances, tumors can develop hybrid vascular networks combining angiogenesis and VM to efficiently obtain oxygen and nutrients [25,26]. In addition, structural heterogeneity (mosaic vessels) has also been described in solid tumors, including breast cancer, where a vessel may be lined by ECs in some parts and by tumor cells in others, forming hybrid vascular structures associated with intravasation and systemic dissemination of cancer cells [27]. Since it has been demonstrated that VM can enhance metastasis after an anti-angiogenic treatment [28], research in the VM field will surely improve cancer therapeutics.

## 3. Clinical Relevance of VM in Breast Cancer and Association with Clinicopathological Parameters

There is no doubt that a major drawback of anti-angiogenic treatment is the formation of VM. Indeed, by inducing hypoxia, VM may be favored, which in turn enhances distant metastasis [28,29]. Notably, the angiogenesis inhibitor endostatin readily inhibits proangiogenic factors such as vascular endothelial growth factor (VEGF), fibroblast growth factor 2 (FGF2), matrix metalloproteinases (MMPs), and hypoxia-inducible factor 1-α (HIF1A), blocking endothelial tube formation. However, endostatin does not affect VM-forming cells, which after being exposed to this collagen-derived factor remain fully active and capable to configure vascular channels [14]. Other antiangiogenic factors have shown similar results [15], suggesting a differential response of EC-dependent angiogenesis and cancer-dependent VM channels formation. In addition, as with the well-established relationship between microvascular density and metastasis in invasive breast cancer [30], VM also has been associated with malignant cells dissemination and bad prognosis, including higher recurrence, lower survival, larger tumor size, and poorer differentiation grade [16,25,31,32], linking this feature to a more malignant breast cancer phenotype [25,31,32]. The VM-positivity rate and its impact on clinicopathological parameters and prognosis in breast cancer patients have been largely studied in the last two decades. For instance, the study from Shirakawa K et al. [25] showed that from 331 surgically resected breast cancer specimens, only 26 (7.9%) evidenced the presence of VM. A high proportion of these VM-positive tumors exhibited pseudo-comedo formations, which are channels containing blood cells instead of necrotic tumor cells. Notably, in these 26 cases, patients were more likely to have hematogenous recurrence and lower percentage of 5-year survival [25]. However, in another study involving eight clinical reports with 1238 breast cancer patients, the VM-cases rate was higher, specifically 24%, and this was associated with larger tumor size (>2 cm), lymph node metastasis, poorer differentiation grade (grades 2 and 3), and shorter overall survival than those without VM, corroborating that this feature is associated with more aggressive breast cancer tumors and poorer prognosis [32]. 

Interestingly, in invasive ductal carcinoma samples, VM was detected in 13.3% of the analyzed tumors. Still, in this VM-positive group, 75% were significantly associated with bad clinicopathological characteristics, including axillary lymph node metastasis (95.6%), tumor size larger than 3 cm (56.25%), higher histological grade (stage 3, 75%), and overall poor prognosis [33]. Similarly, another study showed that breast cancer patients with VM-positive tumors were related to positive nodal status and advanced clinical stage, being the majority of VM-cases in progressive stage 2 and 3, thus, again, associating VM to a poorer outcome [34]. Of note, a meta-analysis on the role of VM in cancer progression and its prognostic value was undertaken considering different types of tumors, corroborating that the presence of VM predicts poorer survival outcomes in cancer patients [35].

## 4. Relationship between VM and Tumor Phenotype

Human breast cancer tumors are classified mainly considering clinic and histopathologic features, as well as molecular markers. Regarding this, the vast majority of these tumors belong to a group that expresses estrogen receptor alpha (ERα) and progesterone receptor (PR). Tumors overexpressing epidermal growth factor receptor 2 (HER2) generally lack ER and PR, while those that do not express neither of these three proteins are collectively called TNBC tumors. HER2 and TNBC are commonly considered as the most aggressive phenotypes of breast cancer. 

The association between VM and breast tumor phenotype has been investigated. In vitro studies have shown that TNBC aggressive cells are particularly prone to form tubular structures, in contrast to more differentiated breast cancer cells. For example, the TNBC MDA-MB-231 and HCC1937 cells readily formed tubular-like structures in Matrigel [36,37]. In comparison, the ERα-positive cell line MCF-7 has been reported to be incapable of forming VM in this matrix [36]; however, in the presence of some VM drivers such as interleukin 1β, MCF-7 cells formed microvessel-like intersections and cords [38]. Further studies are needed to corroborate the effects of VM-drivers upon tubular-like structure formation in ER-positive breast cancer cells. 

The link between VM and a more malignant breast cancer phenotype is coherent with the previously discussed association between VM and poor prognosis, as well as with the stemness features and increased plasticity characterizing cells with high VM-forming potential [18,39]. Indeed, some stemness markers have been negatively related to the hormone receptor status, while their expression has been found significantly increased in TNBC [18,39]. There are important features of the genotypic and phenotypic differences in breast cancer that confer a greater capacity to develop VM, like in TNBC compared to hormone receptor-positive or HER2-positive tumors. For instance, BRCA1 mutations have been shown to predispose for the basal-like/TNBC tumor subtype [40].

On the other hand, there is also solid evidence showing a positive association between VM and the overexpression of HER2. In a study using the MCF-7 cells, forced exogenous HER2 overexpression allowed these cells to form vessel-like structures in Matrigel, a characteristic previously absent in the parental cell line. Interestingly, this process was associated with increased VE-Cadherin protein expression, which abundance and interaction with the epithelial cell kinase 2 (EPHA2) are known to be linked to VM induction [34,41]. Strongly supporting these observations, studies undertaken in aggressive melanoma cells have shown that knockdown or downregulation of VE-cadherin, EPHA2, or laminin subunit gamma 2 (LAMC2) results in abolishing of their ability to form VM [42,43,44]. Notably, in invasive breast carcinoma specimens, HER2 overexpression highly correlated with VM, further corroborating the in vitro results in MCF-7 cells [33,34]. However, other studies have not found a statistically significant association between HER2 overexpression and VM [32]. The reason for this discrepancy is not known, but may be related to an incomplete transformation to a full vasculogenic phenotype in HER2-positive cells, probably due to a lesser level of aggressiveness or the development of alternative survival pathways not related to HER2. Supporting this hypothesis, it is known that HER2-positive tumor cells previously treated with trastuzumab express antigens normally associated with endothelial and stemness phenotypes, together with VM markers, indicating that the treatment may induce VM. However, and interestingly, these cells were not able to form VM structures unless they had fully developed resistance to trastuzumab. Indeed, trastuzumab-resistant cells readily formed tubular structures on Matrigel, which suggested that while HER2-positive cells remain sensitive to treatment, an incomplete vasculogenic phenotype prevails, while fully resistant cells have already experienced a complete transformation and therefore can form VM channels [45].

## 5. Drivers of Vascular Mimicry in Breast Cancer

Many drivers of VM have been described, but in general, these are factors associated with the epithelial-to-mesenchymal transition (EMT) and stemness acquisition processes. In breast cancer, EMT has shown to be important for stem cell-like characteristics acquisition and maintenance, resulting in VM development [46]. Particularly in patients with TNBC tumors, cancer stem cells are considered the source of VM [19]. Among the stemness markers, CD133 and aldehyde dehydrogenase 1 (ALDH1) are closely related to VM formation [18,39]. In this regard, Liu and collaborators found that CD133-positivity displayed in holoclones of the TNBC cell line MDA-MB-231 correlated with VM-forming capacity and self-renew potential. Notably, these holoclones also expressed ECs markers such as VE-Cadherin, MMP2 and MMP9, demonstrating that CD133-positive cancer stem cells contribute to VM in TNBC by inducing transdifferentiation [19]. 

On the other hand, some tumor microenvironment-derived factors associated with EMT promotion are known to induce VM as well, like the cytokine transforming growth factor beta (TGFB) and the transcription factor TWIST1 [17,47]. In hepatocellular carcinoma, it is known that TGFB promotes VM in vitro and in vivo by inducing VE-Cadherin, MMP2, and LAMC2 [47]. Even though this cytokine has not yet been described as a VM-driver in breast cancer, in mice carrying TNBC xenografts the hypoxia-dependent induction of TWIST1 (a known target of TGFB) increased CD133 positivity, causing resistance to sunitinib treatment due to VM development [17]. In addition, and as previously discussed, aberrant extra-vascular expression of VE-Cadherin has been tightly associated with VM formation in cancer cells, a process thought to be related to the acquisition of an undifferentiated embryonic-like phenotype and possibly to a mesenchymal-to-endothelial transition that renders cancer cells able to form vessel-like structures. Speculatively, this would imply the loss of some mesenchymal markers and the gain of endothelial ones, such as vimentin and VE-Cadherin, respectively.

As mentioned earlier in this review, hypoxia, either as a result of an antiangiogenic treatment or induced naturally in tumor core niches, is involved in VM-formation, and therefore should be considered as an important driver by itself. Indeed, a hypoxic environment causes that tumor cells and those from the microenvironment increase their production of factors involved in VM, such as VE-Cadherin, VEGF, MMPs, TWIST1, and HIF1A [17,48,49,50]. Indeed, HIF1A starts multiple signaling cascades resulting in VM induction, as shown in the MDA-MB-231 and MCF-7 breast cancer cell lines [51]. 

Five years after the first report of VM in breast tumors by Shirakawa [24], Basu G.D. and colleagues reported the involvement of Cyclo-oxygenase (COX)-2 as a driver of VM in breast cancer [9]. They found that the invasive MDA-MB-231 and MDA-MB-435 cells overexpressing COX-2 were able to form VM-channels on Matrigel, while non-invasive MCF-7 and ZR-75-1 expressing null or low COX-2 were unable to do so. Moreover, by using the COX-2 inhibitor celecoxib, the authors concluded that the COX-2-dependent induction of VM implicated pathways related to angiogenesis, proliferation, apoptosis, and cell cycle. In a similar manner as the in vitro results, vascular channels were frequently observed in high grade invasive breast ductal carcinoma overexpressing COX-2, but not in low-grade breast tumors, whereas tumor-bearing mice treated with celecoxib corroborated in vitro results [9]. A decade later, Majumder M. and collaborators linked COX-2 expression in breast cancer cells to the induction of stemness, which is a hallmark of VM. Prostaglandin and the EP4 agonist PGE1OH, acting through the prostaglandin E-2 receptor EP4, upregulated NOTCH/WNT expression via PI3K/AKT signaling pathway, which increased migration, invasion, proliferation, EMT, and spheroid formation. Regarding this, increased ALDH activity was found in COX-2 overexpressing tumorospheres, while COX-2 colocalized with the stemness markers ALDH1, CD44, Catenin, NANOG, OCT3/4, and SOX-2 [52,53]. Altogether, the implication of COX-2/prostaglandin signalization in VM formation by highly aggressive breast cancer cells opens new avenues for the use of COX-2/EP4 as a therapeutic target in breast cancer.

Another known player in VM development is sphingosine-1 phosphate receptor 1 (S1PR1), a bioactive signaling lipid regulating vascular development, function, and maturation. However, its participation in this process is more as an “anti-driver”, since its suppression impairs angiogenesis but contributes to VM generation as well as the promotion of invasion and metastasis [54]. Indeed, a recent study demonstrated that S1PR1 deficiency or knockdown contributed to the generation or increase of VM. This was attributed to the S1PR1-dependent promotion of VE-Cadherin phosphorylation, leading to its separation from β-catenin. Interestingly, the survival analysis suggested that in non-TNBC, S1PR1 significantly correlated with poor patient survival, warranting further studies [54].

A very interesting study published in 2015 by Wagenblast E. and colleagues, clearly identified the contribution of two anticoagulant secreted proteins in driving VM in breast cancer cells in vitro and in vivo. These proteins were Serpine2 and Slpi, which were overexpressed in clones from a heterogeneous population of breast cancer cells that could efficiently enter the vasculature and form lung metastasis [55]. The authors were able to prove that the enforced expression of Serpine2 and Slpi in non-intravasating clones efficiently induced in vitro formation of tubular structures in cells previously incapable of forming VM. They concluded that the expression of Serpine2 and Slpi was “sufficient and necessary” to program breast cancer cells for VM, as if this combination worked as a vasculogenic inductive cocktail. Moreover, due to their anticoagulant properties, Serpine2 and Slpi seemed to promote both the passage of erythrocytes into the tumor as well as that of cancer cells into the bloodstream [55]. In accordance with the tumor phenotypes mostly associated with VM, Serpine2 and Slpi were significantly more expressed in HER2+, TNBC (basal) and claudin-low tumors of relapsing patients [55].

TNBC has been described as the breast cancer subtype with the highest rate of tumor infiltrating lymphocytes [56]. Although in the clinic the presence of these cells has been associated with a more favorable prognosis due to their ability to synergize chemotherapy [56], they also secrete a variety of chemokines and cytokines to the tumor microenvironment that may contribute to the oncogenic process. Regarding this, interleukin (IL)-6, signaling through the signal transducer and activator of transcription 3 (STAT3), has shown to promote tube formation by tumor cells in vivo and in vitro by upregulating VE-Cadherin expression and MMP2 activity [57]. Particularly in breast cancer, other inflammatory cytokines have also proven to be drivers of VM, for instance IL-1β and IL-8. On this subject, it is known that TNBC MDA-MB-231 cells express IL-8 as well as its receptors CXCR1/CXCR2, and notably, it has been shown that IL-8 uptake increases during VM formation and that IL-8/CXCR2 signaling is necessary for tube formation, a process that correlates with increased IL-8 levels [58]. In a similar manner but using a different signalization pathway, IL-1β has shown to stimulate VM formation by MCF-7 and MDA-MB-231 cells [38]. Indeed, when these cells were incubated in the presence of IL-1β, they readily formed tube-like structures in Matrigel and expressed VM biomarkers, including VE-Cadherin, VEGF receptor-1, MMP-9, and MMP-2. Of note, this effect was preserved under both normoxic and hypoxic conditions and involved the p38/MAPK and PI3K/Akt signaling pathways [38].

Additionally contributing to VM induction is the BRCA-human chorionic gonadotropin (hCG) axis. The BRCA1 protein is involved in DNA repair mechanisms, and it has been demonstrated that BRCA1-deficient mouse mammary tumors are enriched in CD44+/CD24^−/(low)^ and CD133+ cells. These highly tumorigenic cells show expression of stem cell-associated genes such as *OCT4, NOTCH1, ALDH1, FFGR1, SOX1* [59]. On the other hand, an inverse correlation between BRCA1 and hCG has been found. Indeed, BRCA1 directly represses the expression of β-hCG by binding to its promoter [60]. This hormone is known to exert a potent proangiogenic effect on hCG/luteinizing hormone receptor (hCG/LH-R)-expressing uterine ECs. Moreover, it has been reported that hCG-secreting tumors promote neovascularization and capillary sprouting on in vitro models [61]. Interestingly, mutated BRCA1 in breast cancer cells is associated with β-hCG overexpression, which results in pluripotency and EMT. Besides, this correlated with enhanced migration, invasion, and greater tumorigenic capacity along with expression of EMT and stem cell markers [60]. Notably, all the cellular processes aberrantly activated in BRCA1-mutated cancers are closely related to the VM capacity of tumors, and it has been documented that hCG is crucial for the transdifferentiation of cancer cells into endothelial-like cells by inducing expression of ECs markers such as CD31 and VEGF among others [62]. Remarkably, in breast cancer with mutated BRCA1, β-hCG can signal through transforming growth factor beta receptor II (TGFβRII) regardless of the hCG/LH-R status, resulting in increased cell proliferation [60]. The activation of the TGFB signaling pathway also induces the expression of Snail, Slug, TWIST, and ZEB-1, which in turn increase the expression of mesenchymal markers leading to EMT, a well-known driver of VM. Nevertheless, the role of hCG in VM induction may vary depending on the tumor phenotype. For instance, in hCG/LH-R-positive luminal-A breast cancer cell lines, hCG inhibited cell proliferation and tumor growth [63], whereas, in HER2 positive breast cancer cells, hCG enhanced growth and metastasis in vivo [64]. Therefore, the subtype of breast cancer should be taken into consideration for a clinical approach targeting hCG. 

Another important axis involved in VM is the leucine rich repeats and immunoglobulin like domains 1 (LRIG1)-HER2 axis. LRIG1 is a tumor suppressor that negatively regulates tyrosine kinase receptors (TKRs) signaling by inducing their degradation via ubiquitination and/or hindering the TKRs heterodimeric conformation. This results in the inhibition of PI3K/AKT and ERK1/2 signaling pathways [65]. Among the TKRs regulated by LR1G1 is HER2, which, as previously discussed in this review, has important relevance on the processes associated with the VM capacity of HER2-enriched breast cancer cells. In addition, in other breast tumor subtypes, including the TNBC, LRIG1 expression is known to be decreased, a clinical feature associated with decreased relapse-free survival, higher-grade tumors, and EMT activation [66,67]. Conversely, restoration of LRIG1 expression provokes a mesenchymal-to-epithelial transition, as well as loss of tumorigenic and invasiveness potentials of highly invasive basal breast cancer cells [68]. Interestingly, a proposed molecular mechanism involved in the down-regulation of LRIG1 in breast cancer is mediated by HER2 itself. Indeed, a study from 2008 showed that HER2-induced mammary tumors in transgenic mice had significantly suppressed LRIG1 protein levels, and the activation of HER2 induced a further dramatic loss of endogenous LRIG1 expression and enhancement of proliferation via Akt/Erk, showing that HER2 oncogenic signaling actively contributes to suppression of LRIG [66]. In contrast, LRIG1 gene expression was found enriched in ERα-positive breast cancer, and consistently, LRIG1 has proven to be a transcriptional target of ERα. Moreover, LRIG1 restricts estrogen-driven tumor cell growth, suggesting that it can suppress ERα-positive tumors [67]. This might explain why ERα-positive breast cancer has a lower incidence of VM compared to HER2 and TNBC.

## 6. VM Regulation by Noncoding RNAs in Breast Cancer

Noncoding RNAs (ncRNAs), such as microRNAs (miRNAs) and long noncoding RNAs (lncRNAs), are involved in VM regulation and tumorigenesis of breast cancer. Herein, we review some ncRNAs known to be involved in this process.

The miRNA-299-5p is downregulated in cell lines and tumor and serum samples from breast cancer patients [69,70], and the restoration of its expression inhibited cell migration, invasion, and metastasis [70]. Interestingly, this miRNA is also critical for the development of vascular-like structures by regulating *de novo* expression of osteopontin, which plays a critical role in the VM process of spheroid-forming cells in breast cancer [71]. Another ncRNA involved in breast cancer progression is the tumor-suppressive miRNA-193b [72]. It has been demonstrated that this molecule regulates VM by targeting the dimethylarginine dimethylaminohydrolase 1 (DDAH1) enzyme involved in the metabolism of asymmetric dimethylarginine and monomethyl arginine that are inhibitors of nitric oxide synthesis. Ectopic expression of miR-193b reduced DDAH1 expression and completely inhibited tube formation in MDA-MB-231 cells [73].

P73 antisense RNA 1T (TP73-AS1) is a lncRNA that promotes breast cancer cell invasion and migration [74,75]. In TNBC, TP73-AS1 also participates in VM formation since it decreases miR-490-3p levels implicated in the negative regulation of the TWIST1 gene, which participates in EMT promotion and VM formation [76].

The miR-204 is a tumor suppressor down-regulated in breast cancer and associated with poor prognostic [77]. An overall survival analysis of 3951 breast cancer patients indicated that low miRNA-204 and high FAK/SRC levels were associated with low overall survival of patients. Interestingly, ectopic restoration of miR-204 in MDA-MB-231 cells produced a potent inhibition of VM by reducing the number of branch points and patterned 3D channels. This was associated with the downregulation of several transducers involved in the activation of PI3K/AKT, RAF1, MAPK, VEGF, and FAK/SRC signaling [78]. 

HOX transcript antisense RNA (HOTAIR) is a lncRNA that sponges tumor-suppressive miRNAs. Interestingly, knockdown of HOTAIR resulted in an increment of miR-204 levels, as well as the reduction of migration and hypoxia-induced VM formation by targeting the FAK signaling in TNBC cells [79].

The miR-126-3p expression was significantly downregulated in TNBC cells, where its overexpression inhibited cell proliferation, migration, invasion, colony formation capacity and VM by targeting the regulator of G protein signaling 3 (RGS3), which promotes these processes [80].

As discussed earlier in this review, IL-6 signaling is implicated in chemoresistance and metastasis of various tumors, including breast cancer [81,82,83]. Interestingly, cisplatin treatment upregulated IL-6 levels in ECs, and the resulting conditioned medium induced VM formation in MDA-MB-231 breast cancer cells that might eventually promote drug resistance and metastasis. The mechanism that contributes to VM implicates miR-125a and let-7e downregulation in response to cisplatin treatment, affecting the IL-6 pathway due to IL-6 targeting by these miRNAs, as well as the IL-6 receptor and the STAT3 genes in ECs [84]. Another known miRNA involved in hampering IL-6-stimulated VM in vitro and in vivo is miR-29b, which represses the expression of STAT3 and MMP2 by directly binding to the UTRs of their mRNAs [57].

The signaling of AXL receptor tyrosine kinase promotes cancer stem cell-like phenotypes, drug resistance, metastasis, and EMT. Overexpression of this receptor promotes the regulation of VM formation in breast cancer cells [73] through miRNA-34a that targets the 3’-untranslated region (UTR) [85,86]. In this sense, it was demonstrated that miRNA-34a overexpression downregulated AXL receptor expression resulting in the inhibition of VM formation, migration, and invasion in MDA-MB 231 cells [28].

The miR-93 levels are enriched in TNBC tissue, which has been associated with the occurrence of EMT and VM formation. Accordingly, knockdown of this miRNA resulted in an increase of E-Cadherin and Occludin gene expression and reduction of Vimentin and N-Cadherin levels, as well as the decrease in microtubule forming ability by MDA-MB-231 cells [87,88]. Likewise, forced expression of miR-93 in MT-1 human breast carcinoma cells resulted in tumors containing more blood vessels than those formed by non-miR-93 expressing cells. Accordingly, the expression of miR-93 promoted tumor cell metastasis to lung tissue. It was concluded that the potential target mediating miR-93′s effects was the large tumor suppressor, homolog 2 (LATS2). Indeed, increased expression of LATS2 was associated with tumor cells death and decreased cell survival and invasion [89].

The ncRNAs represent an attractive approach in cancer since they may be considered biomarkers associated with tumors’ biological and clinical characteristics with an important diagnostic and prognostic value. Their implication in VM formation may provide the theoretical basis for anti-vascular therapy in human TNBC as therapeutic targets to inhibit tumor neovascularization.

## 7. Targeting Microenvironment to Overcome VM in Breast Cancer

As discussed earlier in this review, many factors deriving from the tumor microenvironment are involved in VM induction. Likewise, resident cells such as lymphocytes, macrophages, fibroblasts, and tumor cells themselves may produce VM-promoting factors including the inflammatory cytokines IL-6, IL-8, TGFB, and IL-1β. Therefore, the signaling pathways associated with these cytokines offer potential oncological targets to design therapeutic strategies aimed to control the aggressiveness of breast cancer tumor cells. In this regard, since aberrant TGFB signaling is of primordial importance for VM induction, this pathway offers a good opportunity for a VM-targeted therapy. Indeed, TGFB from the tumor microenvironment significantly stimulates tumor growth, migration, invasion, and angiogenesis, which results in an overall poor prognosis. Conversely, blockade of this signalization pathway has been associated with significant inhibition of human basal-like breast cancer metastasis [90], while TGFB-targeted clinical/preclinical studies in breast cancer have shown delayed tumor growth [91]. Furthermore, TGFB inhibition has been shown to enhance chemotherapy action against TNBC [92]. All considered TGFB-targeting warrants further studies for VM inhibition.

The participation of platelets in tumorigenesis and metastasis is a well described process; however, only recently the inhibitory effect of platelets on VM formation by breast cancer has been revealed [93]. Indeed, the ability of HS-578T and MDA-MB-231 TNBC cells to form VM structures on Matrigel was significantly inhibited by their coculture with platelets, while already existing VM structures were readily disassembled by these clotting agents as well. This anti-VM capacity was attributed to the release of soluble factors from the platelets, opening new avenues for further studies aimed to identify these factors in order to target VM-formation.

On the other hand, and as described before, phenotype-related signaling pathways could be useful for targeting microenvironmental changes involved in VM development. This is related especially with modifications on the ECM and plasticity involved in EMT, transdifferentiation, and stemness. A clear example is the use of inhibitors against heat shock protein of 90 kDa (Hsp90). This is a subfamily of molecular chaperones that regulate folding, unfolding, activation, degradation, and intra- or extracellular localization of more than 200 proteins named “client proteins” such as hormone receptors and several kinases [94]. As a chaperone, Hsp90 can rescue functionality on mutated client proteins that would be degraded or inactivated in its absence. In evolution, this increases genetic diversity [95]; however, in cancer, this can lead to the up-regulation of signaling client proteins involved in carcinogenesis, invasion, or metastasis. Therefore, Hsp90 functional inhibition has shown promising effects by degradation of client proteins and shutdown of the processes involved in tumor progression [96]. Particularly in BRCA1-deficient mice, spheroid-forming cells resistant to DNA-damaging drugs could be efficiently re-sensitized by the Hsp90 inhibitor 17-DMAG [59]. Furthermore, this same strategy but with Geldanamycin, an antitumor antibiotic that inhibits Hsp90 function by binding to its ADP/ATP-binding pocket, successfully suppressed breast cancer stem cell population in mammospheres, along with proliferation and migration [97]. One of the possible mechanisms involved in such effects is the regulation of MMP-2 and MMP-9 by cytosolic isoforms Hsp90α and Hsp90β since it has been demonstrated that the interaction between Hsp90 and MMP-2/9 is necessary for its secretion and activation [98]. Taken together, Hsp90 inhibition might represent a highlight in the regulation of the microenvironment factors that trigger VM in breast cancer.

Another strategy to take into account is the targeting of β-hCG, due to its immune suppressor functions and its vasculogenic effects on BRCA1 defective tumors; however, as mentioned above, this is highly dependent on the molecular characteristics of patients [60], warranting further studies.

## 8. Conclusions

**Figure 3:**
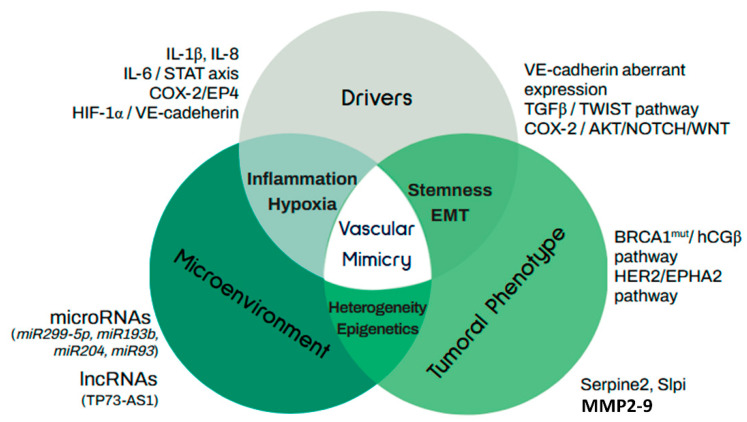
Molecular regulators of vascular mimicry. Vascular mimicry in cancer is activated by several drivers associated with phenotypic features of tumors and microenvironmental factors and signals. Tumor phenotype relates to stemness and EMT capacity of cancer cells. Molecular drivers include TGFB/TWIST regulation of the stem cellular subpopulation and the aberrant expression of VE-cadherin, while the COX-2 axis regulates stemness and VM capacity by PI3K/AKT activation and induction of NOTCH/WNT expression. In the context of breast cancer, the main mechanisms associated with stemness are mediated by hCGβ in BRCA1 mutated patients and by EPHA2 in HER2+ tumors. Heterogeneity in breast cancer tumors is associated with the activation of VM and metastasis through the expression of anti-coagulant factors Serpine2 and Slpi. Along with this, highly invasive breast tumors overexpress metalloproteinases as MMP2 and MMP9, which participate in VM by remodeling the extracellular matrix. Regarding epigenetic regulation of VM, ncRNAs, including several microRNAs as well as lncRNA participate in this process. Well-known microenvironment factors and drivers inducing VM include IL-1β, IL-8, and IL-6. Finally, HIF1A activation in response to hypoxic conditions is highly involved with VE-cadherin expression through TWIST1.

In cancer, VM is an alternative survival strategy adopted by cancer cells under hypoxic stress that allows them to adapt, grow, and disseminate and that is significantly related to poor prognosis and adverse clinicopathological parameters. Besides hypoxic stress, other drivers may also initiate this process, including antiangiogenic therapies and molecules derived from the microenvironment. In breast cancer, VM has been generally associated with HER2-positive and triple-negative breast tumors, as well as with stemness and EMT markers. A scheme summarizing the molecular regulators of VM as well as its relationship with stemness and prognosis is provided in Figure 3.

It is of paramount importance to understand VM biology in order to efficiently target its drivers and to avoid its consequences.

## Figures and Tables

**Table 1:** Distinctive features/markers between vasculogenic mimicry and angiogenesis.

Vasculogenic Mimicry	Angiogenesis	References
Formation of vascular channels from cancer stem cells (tumor cells).	Development of new blood vessels and capillaries from pre-existing ones.	[1,12]
Patterned networks of interconnected loops and cords formation	Formation by sprouting or intussusception	[1,7]
Formed by tumor cells and cancer stem cells	Formed by endothelial cells	[7]
Aberrant expression of VE-Cadherin	VE-Cadherin localization in cell membranes	[13]
PAS+, CD31^−/low^ staining	PAS^−/low^, CD31+ staining	[7]
Factor VIII-related antigen negative or low	Factor VIII-related antigen highly positive	[7]
Unaffected by endostatin and other antiangiogenic factors	Inhibited by antiangiogenic factors	[14,15]
EPHA2, TIE1, LAMC2, overexpression	EPHA2, TIE1, LAMC2 generally negative.	[16]
Express stemness markers, e.g., CD133, ALDH1	CD133 positivity mostly in endothelial precursor cells	[17,18,19,20]
More abundant in poorly differentiated tumors, such as HER2+ and TNBC	Present in embryogenesis, wound healing and tumor growth	[16,21]

## Data Availability

Not applicable.

## Abbreviations

DDAH1	Dimethylarginine dimethylaminohydrolase 1
ECM	Extracellular matrix
ECs	Endothelial cells
EMT	Epithelial-to-mesenchymal transition
hCG	Human chorionic gonadotropin
HOTAIR	HOX transcript antisense RNA
IL	Interleukin
LATS2	Large tumor suppressor, homology 2
LH	Luteinizing hormone
LHCGR	Luteinizing hormone/chorionic gonadotropin receptor
lncRNAs	Long noncoding RNAs
LRIG1	Leucine rich repeats and immunoglobulin like domains 1
miRNAs	MicroRNAs
ncRNAs	Noncoding RNAs
PAS	Periodic acid-Schiff
RGS3	G protein signaling 3
RR	Relative risk
S1PR1	Sphingosine-1 phosphate receptor 1
STAT3	Signal transducer and activator of transcription 3
TNBC	Triple-negative breast cancer
TKR	Tyrosine kinase receptor
TP73-AS1	P73 antisense RNA 1T
UTR	Untranslated region
VM	Vasculogenic mimicry
